# Antiadipogenic and antiobesogenic effects of pterostilbene in 3T3-L1 preadipocyte models

**DOI:** 10.55730/1300-0152.2649

**Published:** 2023-02-21

**Authors:** Birgül GÜLNAR, İpek CANATAR, Sibel ÖZDAŞ

**Affiliations:** Department of Bioengineering, Faculty of Engineering Sciences, Adana Alpaslan Türkeş Science and Technology University, Adana, Turkey

**Keywords:** Antiadipogenic, antiobesogenic, pterostilbene, 3T3-L1, adipogenesis, differentiation, *glucose transporter-4*, *adiponectin*

## Abstract

Since obesity causes at least 2.8 million death each year and is a major risk factor for many diseases, it is critical to evaluate alternative treatment approaches. In this context, studies on the research of natural product-based therapeutics in the fight against obesity are increasing.

In this study, it was aimed to evaluate the antiadipogenic and antiobesogenic efficacy of pterostilbene a natural phenolic compound in 3T3-L1 cells.

The mature 3T3-L1 adipocytes were exposed to pterostilbene at different concentrations and half-maximum inhibitory concentrations (IC_50_) were determined by MTT assay. Oil-Red-O staining was applied to determine lipid accumulation. Phase contrast microscopy, Giemsa, F-actin and DAPI staining were applied to examine the efficacy of pterostilbene on the morphology of 3T3-L1 adipocyte cells. Moreover, expressions of *adinopectin* and *glucose transporter-4 (Glut-4)* in relation to insulin resistance were evaluated using immunofluorescent staining and qRT-PCR.

Pterostilbene caused no significant cytotoxicity towards preadipocytes at concentrations ≤7.5 −M and the percentage of viable cells remained above about 86% for 24 h, 48 h and 72 h (p > 0.05). Therefore, pterostilbene treatment at 5 and 7.5 −M was used in the subsequent experiments as safe dosages. In addition, it was observed that pterostilbene treatment reduced lipid accumulation in adipocyte differentiation. Adipocytes treated with a dose of 7.5 −M for 14 days showed less intense lipid deposition and a more spindle-like morphology compared to the adipocytes treated with a dose of 5 −M. Especially on the 14th day, actin filaments were filamentous in adipocytes treated with pterostilbene 7.5 −M compared to the adipocytes treated with a dose of 5 −M; the filaments were similarly oriented as in preadipocytes, and chromatin condensation was observed to be quite high.

Our data suggests that the pterostilbene supplementation may help weight control and the antiadipogenic and that antiobesogenic activity is mediated in part by reduction of lipid accumulation and induction of *Glut-4* and *Adiponectin* levels.

## 1. Introduction

Obesity is one of the most prominent health problems in the world, and is the fifth leading risk factor for global deaths (The European Association for the Study of Obesity, 2021). Obesity is a chronic metabolic illness caused by an energy imbalance between caloric intake and expenditure (Isganaitis and Lustig, 2005). It is one of the principal risk agents for many illnesses such as hypertension, dyslipidemia, cardiovascular diseases, sleep apnea, infertility, type 2 diabetes, liver metabolic dysfunctions, and cancer (Hurt et al., 2010; Willis et al., 2021).

Excess calories are accumulated in adipocytes as triglycerides, causing the growth and enlargement of white adipose tissue with the process of hypertrophy and hyperplasia (Longo et al., 2016). Adipogenesis is a complex process that involves clonal expansion, differentiation, and intracellular accumulation of triglycerides by preadipocyte cells activated by peroxisome proliferator at the cellular level (Raciti et al., 2018). Adipose tissue formed by adipocytes provides storage of excess fats, as well as performs critical endocrine functions that directly affect insulin sensitivity, which disrupts the overall energy balance and causes various metabolic diseases (Longo et al., 2016).

Along with lifestyle changes in the fight against obesity, pharmacotherapy and bariatric surgery constitute the current therapeutic approaches. However, the development of specific targeted therapeutic molecules and complementary therapy strategies is still a subject of intense research, as current medical and surgical methods involve high risk and cost (Müller et al., 2022).

Nutraceuticals are nutritional products derived from plant and food sources that have medicinal benefits. Data from in vitro, in vivo, and clinical studies have demonstrated the positive effects of nutraceuticals as a complementary strategy in the medical treatment of many diseases, including diabetes and obesity (Peddio et al., 2022). Given the growing trend towards natural product-based therapeutics, the current study was conducted with pterostilbene (trans-3,5-dimethoxy-40-hydroxystilbene; PTS). PTS is a natural phenolic compound and a dimethylated analogue of resveratrol. Furthermore, one hydroxyl and two methoxy groups in the structure of PTS provide more cellular uptake and lipophilic properties with a higher potential than resveratrol, which has three hydroxyl groups in its structure (Langcake and Pryce, 1977; Mattivi et al., 1995). PTS is mainly found in the heartwood of red sandalwood (Spath and Schlager, 1940), blueberries and other berry-like fruits (Aiyer et al., 2012), grapes (Adrian et al., 2000), and the Indian Kino tree (*Pterocarpus marsupium, P. marsupium)* (Fuendjiep, et al., 2002). When the molecular structures of PTS and resveratrol are compared, it has been noticed that they have similar biological properties in antiinflammatory, antioxidant, anticancer, antidiabetic activities and immune regulation activities due to their structural similarities (Bhakkiyalakshmi et al., 2016). *P. marsupium* has long been used in Ayurvedic medicine for its medicinal traits. Its gum resin has been used as the sole herbal product by Ayurvedic practitioners for blood-glucose level reduction (Maurya et al., 2004). The antihyperglycemic activity observed after oral administration of *P. marsupium* tested in rats with streptozotocin-induced hyperglycemia was first demonstrated in 1977 by Manickam et al. (Manickam et al., 1977). It has been reported that PTS treatment significantly prevents hyperinsulinemia and hypertriglyceridemia, and significantly reduces serum glucose level and body fat accumulation (Grover et al., 2005; Nagao et al., 2017).

In this study, the antiadipogenic and antiobesogenic efficacy of PTS in 3T3-L1 preadipocytes were investigated by evaluating cell viability, cell proliferation, lipid accumulation, cell morphology, chromatin condensation, and the expression levels of genes linked to the insulin resistance and adipogenesis.

## 2. Materials and methods

### 2.1. PTS preparation

The range of used PTS concentrations (1–20 −M) was determined based on the cited scientific reports (Hsu et al., 2012; Seo et al., 2017; Bedi et al., 2020). PTS (HPLC purity, 99.8%) was obtained from Sabinsa Corporation. PTS was dissolved in dH_2_O both in 1 M and 1 mM stock solutions and then kept at −20 °C under shade until use.

### 2.2. Cell culture and proliferation

3T3-L1 (CRL-173) preadipocyte cells were taken from the American Type Culture Collection (ATCC, Manassas, VA) and seeded into T25 culture flasks at a density of 1 × 10^6^ cells/cm^2^ containing expansion medium. 3T3-L1 cells expansion medium was prepared in a 90% DMEM medium prepared with FBS to a final concentration of 10% supplemented with 1% L-glutamine and 1% penicillin/ streptomycin. The cells were incubated in a humidified environment at 37 °C 5% CO_2_ and 95% air in an incubator (İldam, ILD-CI-80, Turkey). Culture media was changed every 3 days in a biosafety cabinet (DEMAIR, MSC-II A, Turkey). In addition, images of cells were obtained with an inverted microscope (Leica, Dmil Led Fluo, Thermo Fisher, Germany).

### 2.3. Cells viability assay

MTT[3-(4,5-dimethylthiazol-2-yl)-2,5diphenyl tetrasodium bromide] was used to investigate the effect of PTS on 3T3-L1 preadipocyte viability and to detect the appropriate PTS dose for use in future studies (Seo et al., 2017). The MTT assay was conducted as described in the International Standards Organization (ISO) 10993-5 norms (International Organization for Standardization, 2009). 3T3-L1 preadipocytes were cultured into 96-well plates at a density of 2 × 10^3^ cells/well. The medium was removed when 3T3-L1 preadipocytes reached 80%–90% confluency. The cells were treated with DMEM medium supplemented with various concentrations of PTS (1–2.5–5–7.5–10–12.5–15–17.5–20 −M) and 0.1% (v/v) dimethyl sulfoxide (DMSO) except for the DMEM controls.

For MTT assay, the culture media was aspirated and cells were rinsed with PBS. MTT solution was prepared 0.5 g/mL in PBS and 100 −L of the solution was applied to wells. The cells were incubated at 37 °C for 4 h. Afterwards, 100 −L of DMSO was supplied to the wells for 30 min. The relative cell viability was measured with spectrophotometric (Uvmini-1240, Shimadzu, Kyoto, Japan) optical density (OD) at wavelength of 570 nm. The relative cell-viability was plotted into graphs. Calculation of cell viability percentage was done by using Eq. 1.


(Eq. 1) $$\text{Cell viability }(\%)=(\text{Average }{\text{OD}}_{570}\;\text{of treated cells}/\text{Average }{\text{OD}}_{570}\;\text{of control cells})\times 100$$

### 2.4. Differentiation

The 3T3-L1 preadipocytes were differentiated into mature adipocytes in an adipocyte maintenance medium in the presence and absence of PTS (Mosmann, 1983). The differentiation medium consisted of 90% DMEM, 10% FBS, 1% L-glutamine, 1% penicillin/streptomycin, 1−M dexamethasone (DEX), 0.5 mM isobutylmethylxanthine (IBMX) and insulin (1 −g/mL). First of all, 3T3-L1 cells were cultivated at 1 × 10^6^ in a 25 cm^2^ flask filled with preadipocyte growth media. Preadipocyte cells were induced to differentiate using a differentiation medium modified either with the absence or presence of PTS for 48 h. After that, the medium was aspirated and an adipocyte maintenance medium containing DMEM with 10% FBS and insulin (1−g/mL) was added. An inverted microscope was used to examine differentiated adipocyte cells.

### 2.5. Oil-Red-O staining

Oil-Red-O staining kit was applied to examine and determine the lipid accumulation in 3T3-L1 preadipocytes, differentiated adipocytes and the efficacy of PTS in 3T3-L1 adipocytes (Green and Kehinde, 1975; Hsu et al., 2012). The preadipocytes, differentiated adipocytes, and PTS loaded adipocytes were seeded on coverslips plated in 12-well plate (1 × 10^5^ cells/well) for Oil-Red-O staining. Coverslips were taken at day 14 and the medium was aspirated. Then, cells were gently rinsed with PBS. After that, cells were fixed using 1 mL of 10% (v/v) formalin for 30 min. The cells were then rinsed with distilled water and 1 mL of 60% isopropanol (v/v) was added for 5 min. The cells were stained directly with 1 mL Oil-Red-O solution for 20 min. After incubation, the Oil-Red-O solution was aspirated and cells were rinsed with distilled water until the stain was gone. Hematoxylin was immersed into the wells and cells were incubated for 1 min and rinsed 2–5 times with distilled water. Briefly, images of cells were obtained using a microscope. In addition, the total cell lipid content was measured by washing with isopropanol to remove stain residues. Also, spectrophotometry was employed to measure OD at a wavelength of 500 nm (Nanoprop 2000c, Thermo Scientific, USA) (Green and Kehinde, 1975; Hsu et al., 2012).

### 2.6. Live-dead assay

In order to assess the effectiveness of PTS on cell viability, live-dead cell assay was used (Hu et al., 2018). Ethidium homodimer-1 (Eth-1) binds to the DNA of cells with impaired cell membrane integrity, showing dead cells with red fluorescence. Calcein-AM (C-AM), on the other hand, is a nonfluorescent acetomethoxy-derived compound, but it is broken down by esterase enzymes in the cytoplasm and turns into calcein, which makes living cells appear green with the effect of fluorescence. For the live/dead cell assay, 3T3-L1 preadipocytes, differentiated adipocytes in the presence or absence of PTS were seeded on coverslips into a 24-well plate. The assay was performed on days 0th, 7th and 14th. Firstly, cells were washed twice with 250 −L of +/+ PBS. For the continuation of the staining process, 1 −M Eth-1 and 1 −M C-AM were subjected to the wells for 30 min in the dark. The cells were rinsed by 250 −L +/+ PBS and examined at 10X to assess the distribution of cells on the coverslip with the aid of a fluorescent microscope. After the examinations, images were taken for each coverslip. ImageJ software was used to determine the relative cell viability in each area. Calculation of cell viability (%) was done by using Eq. 2.


(Eq. 2) Viable Cells (%)=[Viable cells/(Viable cells+Dead cells)]×100

### 2.7. Phase contrast microscopy

The 3T3-L1 preadipocytes, 3T3-L1 adipocytes in the presence or absence of PTS were followed and monitored while the cultivation process continued by using phase contrast microscopy (Leica, Dmil Led Fluo, Thermo Fisher, Germany). Since the cells were visible in the plate, different staining procedures were not required. The cells were imaged at day 0, day 7, and day 14 under 10× magnification.

### 2.8. Immunofluorescence staining

Immunofluorescence (IF) staining was applied to examine the effect of PTS on the intracellular level of *Glut-4* and *adinopectin* and cell morphology in 3T3-L1 adipocytes. Immunofluorescence staining was performed on day 0, day 7 and day 14. The 3T3-L1 preadipocytes, differentiated adipocytes, and PTS loaded adipocytes were cultivated into a 6-well plate. Twenty-four hours later, the coverslips were washed with 1X PBS. After the washing process was finished, the coverslips in the 6-well plate were transferred to the 24-well plate, one for each well, with the help of sterile needle and tweezers. For the fixation process, 250 −L of 4% (w/v) paraformaldehyde was applied on the coverslips and incubated at room temperature for 20 min. Then, the paraformaldehyde was discarded from the wells and the coverslips were washed 3 times with cold 300 −L 1X PBS. Permeabilization is a very important process since the target protein is intracellular. Therefore, 250 −L of 0.1% Triton X-100 (thawed in 1X PBS, v/v) was applied on the coverslips and incubated for 15 min at room temperature for permeabilization. The wells were washed for 5 min with 250 −L of 1X PBS thrice. After permeabilization, 250 −L of 1% (w/v) BSA in PBS was applied to each well for 30 min at ambient temperature. Afterwards, 250 −L of 1% (w/v) antibeta actin, 1:100 (v/v) of adinopectin and anti-Glut-4 in PBST (PBS + %0.1 Tween 20) were added to cells overnight in a humidified room at 4 °C. Then, cells were gently washed with PBS. Two hundred and fifty microliters of 1:1000 (v/v) Alexa Fluor 488-conjugated goat antimouse IgG H&L was applied to wells as a secondary antibody and cells were incubated for 45 min at room temperature. Then, the cells were rinsed with PBS thrice. To perform the immunostaining procedure, 250 −L Phalloidin-iFluor 488 Reagent diluted in %1 (w/v) BSA was added into the wells for incubation.

Also, for nucleus staining, DAPI diluted in PBS was added to the wells and was incubated in darkness for 45 min at ambient temperature. After the incubation, the solutions were aspirated and the wells were rinsed 3 times with PBS. Finally, the coverslips, on which all staining procedures were applied, were taken onto the slides with the cellular parts on top for visualization. Three microliters of BrightMount/Plus was added to the coverslips taken onto the slides. The IF staining was visualized at 10× magnifications using a fluorescence microscope and ImageJ software was used to analyses fluorescein signals (imagej.nih.gov) (Derici et al., 2021).

### 2.9. Giemsa staining

Giemsa staining was used to analyse the effect of PTS on cell morphology. The 3T3-L1 preadipocytes and differentiated adipocytes in presence or absence of PTS were seeded on coverslips into a 24-well plate (Hu et al., 2018). The coverslips were taken at day 7 and day 14 for fluorescence staining. The wells were eluted with 250 −L PBS. After that, cells were exposed to 250 −L of 60% (v/v) cold methanol diluted in PBS for 1 h to induce fixation. After that, 250 −L 100% (v/v) methanol was added to wells and incubated for 10 min at room temperature. The wells were rinsed with 250 −L of PBS. 0.1 g of Giemsa dye was dissolved in 10 mL of methanol, kept in hot water for 20–30 min and passed through a 0.22 −m filter. One milliliter of Giemsa was added to cover the surfaces of the cells whose fixation process was completed and incubated for 20 min at room temperature. Finally, the dye solution was discarded and the wells were rinsed three times. Cell morphologies were examined at 10X under an inverted microscope. The ImageJ program (imagej.nih.gov) was employed to measure the mean cell diameter in all three areas in images for three wells.

### 2.10. RNA isolation and real-time RT-PCR

To observe the effect of PTS on insulin resistance and adipogenesis, the expression levels of *Glut-4* and *adiponectin* genes in 3T3-L1 cells was evaluated using real-time PCR. The 3T3-L1 preadipocytes, 3T3-L1 adipocytes in the presence or absence of PTS were cultured into a 6-well plate (3 × 10^5^ cells/well) and were harvested at 14 days after the initiation of differentiation for real-time RT-PCR. The total RNA was isolated with TRIzol reagent (Life Technologies, Rockville, MD, USA). Concentrations of RNA samples were determined using a spectrophotometer (Nanodrop 2000c, Thermo Scientific, USA). A Transcriptor High Fidelity cDNA Synthesis Kit (Roche Applied Science, Germany) was subsequently used to convert RNA to cDNA. One Taq 2X Master Mix with Standard Buffers (New England Bio labs Inc., USA) was used for the amplification of cDNA using the primer pairs synthesized by Sentebiolab (Turkey):

*Glut-4*, 5′-TAGGAGCTGGTGTGGTCAATACG-3′ (forward) and 5′-TAAAAGGGAAGGTGTCCGTCG-3′ (reverse);

*Adiponectin* 5′-TACAACCAACAGAATCATTATGAC GG-3′ (forward) and 5′-GAAAGCCAGTAAATAGAGTCGTTGA-3′ (reverse);

*Glyceraldehyde-3-Phosphate Dehydrogenase (Gapdh)*, 5′-TCAACGGCACAGTCAAGG-3′ (forward) and ‘5-ACTCCACGACATACTCAGC-3′ (reverse).

The GoTaq 2-Step RT-qPCR System (Promega, USA) was used for real-time RT-PCR by Qiagen Rotor-Gene Q (Qiagen, Hilden, Germany). The PCR steps were shown in Table. The comparative threshold cycle CT method (^ΔΔ^CT) was performed for target gene expression analysis and the normalisation of each gene expression result was measured with the *Gapdh* gene expression (Li et al., 2016). The Rotor-Gene Q Software (Qiagen, Germany) was used to obtain qRT-PCR results.

### 2.11. Statistical analysis

Graph Pad Prism software version 8.4.3 (GraphPad Software, San Diego, California, USA) was used for statistical analysis of the data. Data was expressed as mean ± standard deviation (SD). Moreover, each experiment was performed in triplicate. All data was tested for the normal distribution using the Shapiro-Wilk test (Ghasemi and Zahediasl, 2012). Since the data was normally distributed, parametric tests were used. Student’s t-test was employed to determine differences among the groups. For multiple comparisons of results, one-way ANOVA analysis followed by Turkey’s tests were used.

## 3. Results

### 3.1.Effects of PTS on cell viability in 3T3-L1 preadipocytes by MTT assay

**Figure 1:**
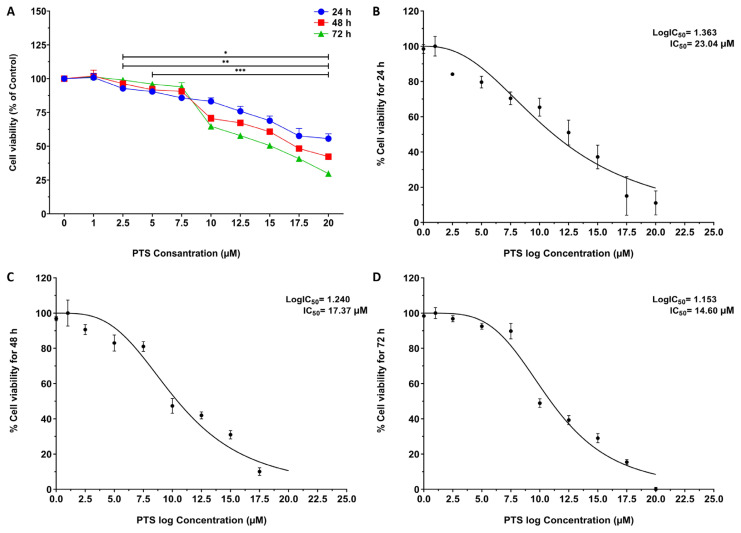
Effect of PTS on the viability of 3T3-L1 cells. **A.** MTT assay data of PTS. 3T3-L1 preadipocytes cultured in 96-well plate were subjected to PTS treatment with increasing concentrations for 24, 48, and 72 h. Cytotoxicity was then calculated by MTT assay. In addition, the cell in the medium without PTS was used as the control and the cell in the medium without PTS but containing 0.01% DMSO was used as the vehicle control. **B. C. D.** IC_50_ values of PTS for 24, 48, and 72 h. IC_50_ was calculated by nonlinear regression model. Data are presented as mean ± SD (n = 3) from three independent experiments and expressed as fold change compared to control. One-way ANOVA test was used for statistical analysis. For 24, 48, and 72 h, respectively; *p < 0.005, **p < 0.005, ***p < 0.005 significantly different from untreated 3T3-L1 preadipocyte. PTS: pterostilbene; MTT: methylthiazole tetrazolium; DMSO: dimethyl sulfoxide; IC_50_: half (50%) maximum inhibition concentration; SD: standard deviation.

Although 3T3-L1 cells doubling time was not reported specifically, it and its subclones had a doubling time of approximately 28 ± 10 h. (https://www.cellosaurus.org/CVCL_0123 https://www.atcc.org/products/ccl-92#detailed-product-information) Also, in MTT assays which are typically performed in two-dimensional cell culture, cells are exposed to the drug for up to 72 h to evaluate the drug cytotoxic efficacy and potency (International Organization for Standardization, 2009; Niepel et al., 2019). Therefore, in order to evaluate the efficiency of PTS on the viability of 3T3-L1 cells and to determine the appropriate dose for use in future studies, the cells treated with PTS at different concentrations between 0 −M and 20 −M for 24, 48 and 72 h were stated as % cell viability along with the results (Figure 1).

At the end of the 24 h incubation, a 10% reduction in the viability of the cells to which PTS concentrations higher than 5 −M was applied compared to the control was observed and the reduction in cell viability continued significantly at higher concentrations (p = 0.6081 for 1 −M; p < 0.0001 for 2.5 −M; p = 0.0001 for 5 −M; p < 0.0001 for 7.5 −M and higher concentrations). After 48 h of incubation, a 10% decrease in the viability of cells treated with concentrations of PTS higher than 7.5 −M was observed compared to the control, and the reduction in cell viability continued significantly at higher concentrations (p = 0.3262 for 1 −M; p = 0.0017 for 2.5 −M; p = 0.0012 for 5 −M; p < 0.0001 for 7.5 −M and higher concentrations). Similarly, after 72 h of incubation, a 10% reduction in viability of cells exposed to concentrations of PTS higher than 8.5 −M was observed compared to control, and the reduction in cell viability continued significantly at higher concentrations (p = 0.2545 for 1 −M; p = 0.0791 for 2.5 −M, p = 0.0005 for 5 −M, p = 0.0073 for 7.5 −M, p < 0.0001 for 10 −M and higher concentrations) (Figure 1A). Moreover, the PTS concentration (IC_50_) causing 50% reduction in viability of cells for 24, 48 and 72 h’ incubation was observed as 23.04 ± 1.89 −M, 17.37 ± 1.22 −M, and 14.60 ± 2.88 −M, respectively (Figures 1B–1D). According to ISO norms, cell viability below 70% compared to untreated control is a sign of cytotoxic effect (International Organization for Standardization, 2009). The data indicated that PTS caused no significant cytotoxicity towards preadipocytes at concentrations ≤7.5 −M for 24 h, 48 h and 72 h (p > 0.05). Moreover, the percentage of viable cells remained above about 86%. However, PTS reduced cell viability below 70%, at concentrations higher than 10 −M for up to 72 h (p < 0.05). Also, in all the evaluated times, the IC_50_ was higher than 14 −M. Therefore, PTS treatment at 5 and 7.5 −M as a safe dose was used in the subsequent experiments. The results demonstrated that the addition of PTS to the medium reduced the proliferation of 3T3-L1 preadipocytes in a time dose dependent manner.

### 3.2. Effects of PTS on terminal differentiation of 3T3-L1 preadipocytes and adipogenesis by Oil-Red-O staining

**Figure 2:**
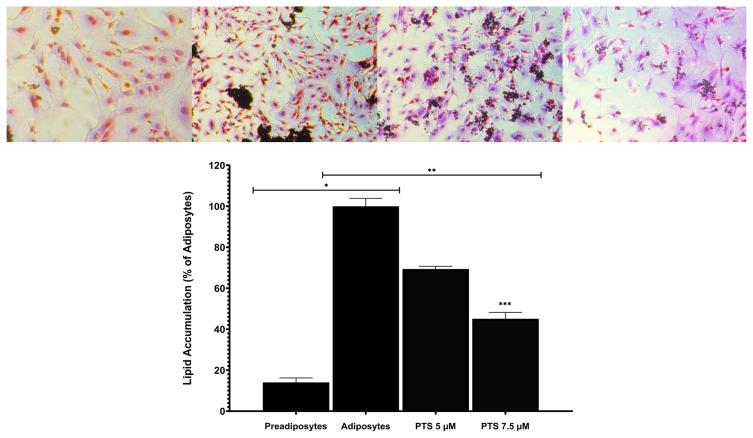
Effect of PTS on adipogenesis and lipid accumulation. **A.** Preadipocytes were differentiated into mature adipocytes for 14 days in the absence of PTS treatment or in the presence of different doses (5 −M or 7.5 −M) of PTS in adipocyte maintenance medium. Undifferentiated and untreated preadipocytes and differentiated and untreated adipocytes were used as controls. Representative microscopic images of cells stained with Oil-Red-O at 10× magnification. **B.** Quantitative data of Oil-Red-O staining. Data with a relative change of OD500 from three independent experiments are presented as mean ± SD (n = 3) and expressed as fold change compared to adipocyte control. One-way ANOVA test was performed for statistical analysis. *p < 0.0001 and **p < 0.0001 differ from untreated 3T3-L1 adipocyte. **p < 0.0001 is significantly different from the 5 −M dose of PTS. PTS: pterostilbene; OD: optical density; SD: standard deviation.

In order to verify the adipocyte phenotype of 3T3-L1 cells and to evaluate the effect of PTS on the terminal differentiation phase of adipogenesis, 3T3-L1 preadipocytes were differentiated into mature adipocytes in an adipocyte maintenance medium in the presence or absence of PTS (5 −M and 7.5 −M) for 14 days. Terminal adipocyte differentiation and lipid accumulation in cells were evaluated by Oil-Red-O staining at day 14 of adipogenesis (Figure 2). In the study, undifferentiated and untreated preadipocytes and differentiated and untreated adipocytes were used as controls. The Oil-Red-O staining intensity in cells examined under an inverted microscope was considerably more intense in untreated adipocytes than in preadipocytes. However, an overall reduction in Oil-Red-O staining was examined in mature the 3T3-L1 treatment adipocyte group treated with different doses of PTS compared to untreated adipocytes. Treatment of cells with a 7.5 −M dose of PTS markedly reduced the level of Oil-Red-O staining compared to the 5 −M dose treatment group of PTS (Figure 2A).

Furthermore, Oil-Red-O dye from 3T3-L1 cells was measured by eluting with isopropanol in order to quantify lipid content in adipocytes. When the lipid accumulations of preadipocytes and untreated adipocytes were compared, an increase of 85.96% ± 2.16% was obtained in untreated adipocytes (p < 0.0001). Comparing the internal lipid accumulation of 3T3-L1 adipocytes, lipid deposition was reduced by 30.64% ± 1.36% in 3T3-L1 adipocytes treated with a 5 −M dose of PTS compared to mature untreated 3T3-L1 adipocytes (p < 0.0001). Intracellular lipid accumulation was reduced by approximately 54.93% ± 3.14% in 3T3-L1 adipocytes treated with a 7.5 −M dose of PTS compared to untreated mature 3T3-L1 adipocytes (p < 0.0001). In addition, lipid accumulation was reduced by 35.02% (p < 0.0001) in adipocytes treated with 7.5 −M dose compared to the 5 −M dose of PTS (Figure 2B). 3T3-L1 preadipocytes were observed to significantly attenuate terminal differentiation in a dose-dependent manner when exposed to PTS, in agreement with other data. These data show that PTS treatment inhibits adipogenesis in 3T3-L1 cells by reducing lipid deposition in adipocyte differentiation.

### 3.2. Effects of PTS on cell viability of 3T3-L1 in adipogenesis by live-dead assay

**Figure 3:**
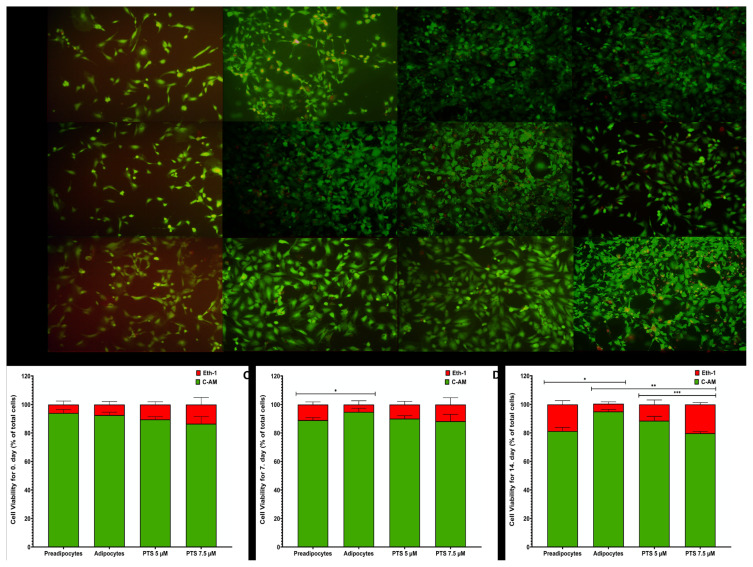
Effect of PTS on the viability of 3T3-L1 cells in the differentiation phase. **A**. Preadipocytes were differentiated into mature adipocytes for 14 days in the absence of PTS treatment or in the presence of different doses (5 −M or 7.5 −M) of PTS in adipocyte maintenance medium. Undifferentiated and untreated preadipocytes and differentiated and untreated adipocytes were used as controls. The microscopic images at 10× magnification of cells stained with C-AM and Eth-1 at day 0, 7 and 14 of differentiation. C-AM and Eth-1 staining showed live and dead cells, respectively. **B. C. D.** Quantitative data of live-dead staining for days 0, 7, and 14 of differentiation, respectively. Three coverslips were evaluated for all cell groups. Six randomly selected regions on each coverslip were visualized microscopically and determined using ImageJ program. Data were stated as the total percentage of live and dead cells showed as mean ± SD (n = 3). One-way ANOVA test was used for statistical analysis. *p < 0.05 and **p < 0.05 differ from untreated 3T3-L1 adipocytes. ***p < 0.05 is significantly different from the 5 −M dose of PTS. PTS: pterostilbene; C-AM: calcein-acetomethoxy; Eth-1: ethidium homodimer-1; SD: standard deviation.

The efficacy of PTS on the viability of 3T3-L1 cells in the differentiation phase was investigated using the live-dead assay. Two different media were investigated for 3T3-L1 cells based on the presence or absence of PTS (5 −M and 7.5 −M) for 14 days, and the cells were differentiated into mature adipocytes in an adipocyte maintenance media. Dual staining was performed on days 0, 7, and 14 to separate live (green) and dead (red) cells using ethidium homodimer-1 (Eth-1) and calcein-acetomethoxy (C-AM), respectively. Representative images of live and dead cells of preadipocytes, adipocytes are demonstrated in Figure 3A.

Under the inverted microscope for day 0 of differentiation, high-intensity green fluorescence and low-intensity red fluorescence were observed in all groups (Figure 3A). The percentage of viable cells for day 0 in preadipocyte and untreated adipocytes were 94% ± 2.40% and 92.50 ± 2.08%, respectively (p = 0.4860). Also, similar to mature 3T3-L1 adipocytes, the percentage of viable cells for day 0 after 5 −M and 7.5 −M treatment of PTS was 89.50% ± 1.90% and 86.50 ± 4.09%, respectively (p = 0.2071 and p = 0.1516). Moreover, the percentage of viable cell change was similar for the 5 −M and 7.5 −M treatments of PTS, with 3.24% and 6.48%, respectively (p= 0.1540) (Figure 3B).

On the 7th day of differentiation, under the inverted microscope, low red fluorescence intensity was observed with high green fluorescence intensity in all groups. However, green fluorescence intensity was lower in untreated adipocytes compared to preadipocytes (Figure 3A). When comparing preadipocytes with untreated adipocytes, it was observed that the viability of untreated adipocytes increased at day 7, and the percentage of viable cells was 89.00% ± 1.82% and 94.75% ± 2.62%, respectively (p = 0.0114). Also, similar to mature 3T3-L1 adipocytes, the percentage of viable cells at day 7 after 5 −M and 7.5 −M treatment of PTS was 90.00% ± 2.16% and 88.25% ± 4.78%, respectively (p = 0.1414 and p = 0.0536). Furthermore, the 5 −M and 7.5 −M treatments of PTS, the percentage of viable cell change was similar. These values were 5.01% and 6.86%, respectively (p = 0.6110) (Figure 3C).

Under the inverted microscope for the 14th day of differentiation, low red fluorescence intensity was observed with high green fluorescence intensity in all groups. However, green fluorescence intensity was lower in untreated adipocytes compared to preadipocytes. Similarly, red fluorescence intensity was partially increased in treatment group adipocytes compared to untreated adipocytes (Figure 3A). Viability increased at day 14 in untreated adipocytes compared to preadipocytes and the percentage of viable cells was 81.25% ± 2.62% and 95.50% ± 1.41%, respectively (p = 0.0005). Also, unlike mature 3T3-L1 adipocytes, the percentage of viable cells at day 14 after 5 −M and 7.5 −M treatment of PTS was 88.50% ± 3.10% and 79.75% ± 1.25%, respectively (p = 0.0319 and p = 0.0008, respectively). Therewithal, the 5 −M and 7.5 −M treatments of PTS, the percentage of viable cell change was different from each other. These values were 6.84% and 16.05%, respectively (p = 0.6110) (Figure 3C).

### 3.3. Effects of PTS on cell morphology of 3T3-L1 in adipogenesis by phase-contrast microscopy, Giemsa staining and immunofluorescence staining

**Figure 4:**
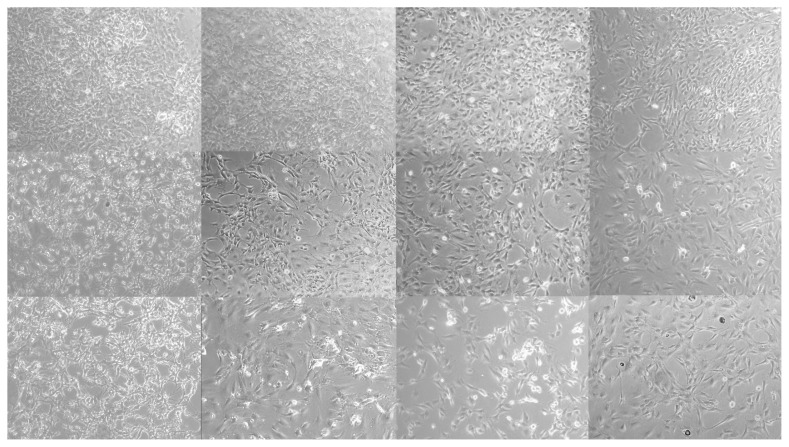
Phase-contrast microscopic images of 3T3-L1 cells in the differentiation phase. Preadipocytes were differentiated into mature adipocytes for 14 days in the absence of PTS treatment or in the presence of different doses (5 −M or 7.5 −M) of PTS in adipocyte maintenance medium. Undifferentiated and untreated preadipocytes and differentiated and untreated adipocytes were used as controls. Representative phase-contrast microscopic images of cells at 0th, 7th, and 14th day of differentiation at 10× magnification.

Adipocyte culture differentiated from 3T3-L1 preadipocytes displays a monolayer of large, spherically shaped cells that partially overlap. Cells were followed using a phase contrast microscope to observe the efficacy of PTS on the 3T3-L1 cells morphology in the differentiation phase. 3T3-L1 preadipocytes were differentiated into mature adipocytes in an adipocyte maintenance medium in the presence or absence of PTS (5 −M and 7.5 −M) for 14 days. Phase-contrast microscopic images of cells the 0th, 7th, and 14th days are presented in Figure 4.

A decrease in mitosis was observed on days 0, 7, and 14 of 3T3-L1 preadipocyte cultures used as controls. In addition, although 3T3-L1 preadipocytes preserved their spindle-shaped morphology, a decrease in density was also observed. However, an increase in mitosis was observed on the 0th, 7th, and 14th days of 3T3-L1 adipocyte cultures, which were used as a control in adipogenesis and differentiated without treatment in an adipocyte maintenance medium. Over time, the cell developed an adipogenic phenotype and acquired a rounded body and a less elongated morphology by lipid accumulation. In the group treated with 5 −M or 7.5 −M doses of PTS in adipocyte maintenance medium, partial adipogenic phenotype cell clumps were observed over time in a time and dose-dependent manner. Adipocytes treated with PTS 7.5 −M on 14 days showed less intense lipid deposition and a more spindle-like morphology compared to the other treatment group.

**Figure 5:**
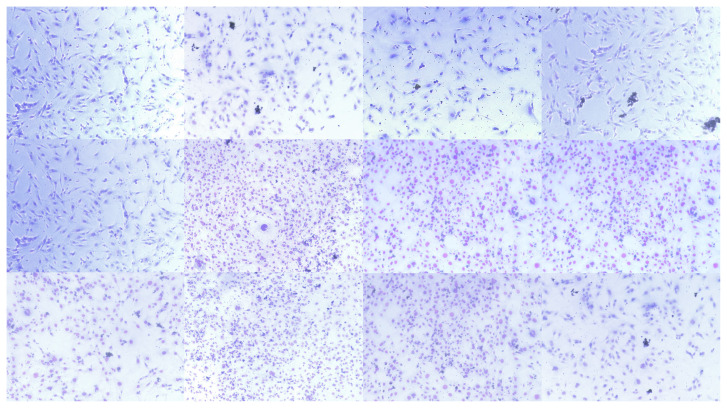
Images obtained through Giemsa staining to show the effect of PTS on the morphological structure of 3T3-L1 cells at the differentiation stage. Preadipocytes were differentiated into mature adipocytes for 14 days in the absence of PTS treatment or in the presence of different doses (5 −M or 7.5 −M) of PTS in adipocyte maintenance medium. Undifferentiated and untreated preadipocytes and differentiated and untreated adipocytes were used as controls. Representative phase-contrast microscopic images at 10× magnification of Giemsa-stained cells at 0th, 7th, and 14th day of differentiation (scale bar: 200 −m).

In Giemsa staining, compared to 3T3-L1 adipocytes differentiated without treatment, 3T3-L1 preadipocytes showed characteristic fibroblastic cell morphology at the 0th, 7th, and 14th days with longer, spindle-like structure and wide spread. Adipocytes differentiated by treatment with 5 −M or 7.5 −M doses of PTS were partially round and small in time and dose-dependent manner. However, especially on day 14, adipocytes treated with PTS 7.5 −M grew dramatically compared to the other treatment group, gaining a long, spindle-like structure as in preadipocytes (Figure 5).

**Figure 6:**
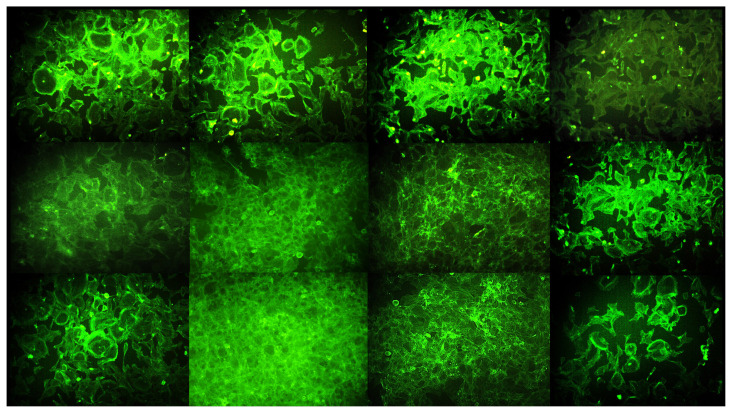
Immunofluorescence staining data showing the effect of PTS on cellular shape and integrity of 3T3-L1 cells in the differentiation phase. Preadipocytes were differentiated into mature adipocytes for 14 days in the absence of PTS treatment or in the presence of different doses (5 −M or 7.5 −M) of PTS in adipocyte maintenance medium. Undifferentiated and untreated preadipocytes and differentiated and untreated adipocytes were used as controls. Representative phase-contrast microscopic images at 10× magnification of cell cytoskeleton stained with Phalloidin (Alexa Fluor-488) at the 0th, 7th, and 14th days of differentiation (scale bar: 200 −m).

In F-actin staining by immunofluorescence, 3T3-L1 preadipocytes exhibited a spindle morphology with multiple cell extensions at the 0th, 7th, and 14th days. In addition, a morphological change from spindle to spherical was observed over time in 3T3-L1 adipocytes differentiated without treatment, and the cells were partially overlapped. Adipocytes differentiated by treatment with 5 −M or 7.5 −M doses of PTS formed cell clusters with a morphology between preadipocytic and adipogenic phenotype in a time and dose-dependent manner. However, compared to the other treatment group and especially on the 14th day, actin filaments were filamentous in adipocytes treated with PTS 7.5 −M, with less integrity and similarly oriented filaments as in preadipocytes (Figure 6).

**Figure 7:**
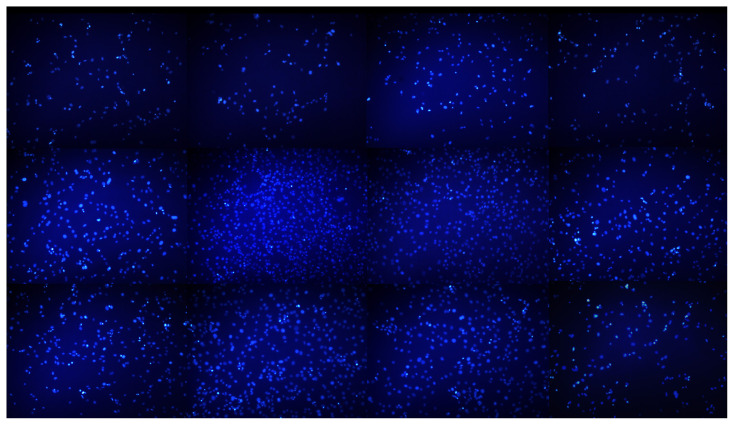
Immunofluorescence staining data showing the effect of PTS on DNA condensation of 3T3-L1 cells in the differentiation phase. Preadipocytes were differentiated into mature adipocytes for 14 days in the absence of PTS treatment or in the presence of different doses (5 −M or 7.5 −M) of PTS in adipocyte maintenance medium. Undifferentiated and untreated preadipocytes and differentiated and untreated adipocytes were used as controls. Representative phase-contrast microscopic images at 10× magnification of DAPI-stained cell nuclei at the 0th, 7th, and 14th days of differentiation (scale bar: 200 −m).

Nuclear staining with DAPI showed increased chromatin condensation, a characteristic of apoptosis over time, to 3T3-L1 preadipocytes compared to 3T3-L1 adipocytes differentiated without treatment. Adipocytes differentiated by treatment with 5 −M or 7.5 −M doses of PTS exhibited slightly increased DNA condensation in a time and dose-dependent manner. However, chromatin condensation was quite high in adipocytes treated with PTS 7.5 −M, as in preadipocytes, especially on day 14 compared to the other treatment group (Figure 7).

### 3.4. Effects of PTS on mRNA level expression of Glut-4 and adiponectin in 3T3 L1 by immunofluorescence staining and RT-PCR

**Figure 8:**
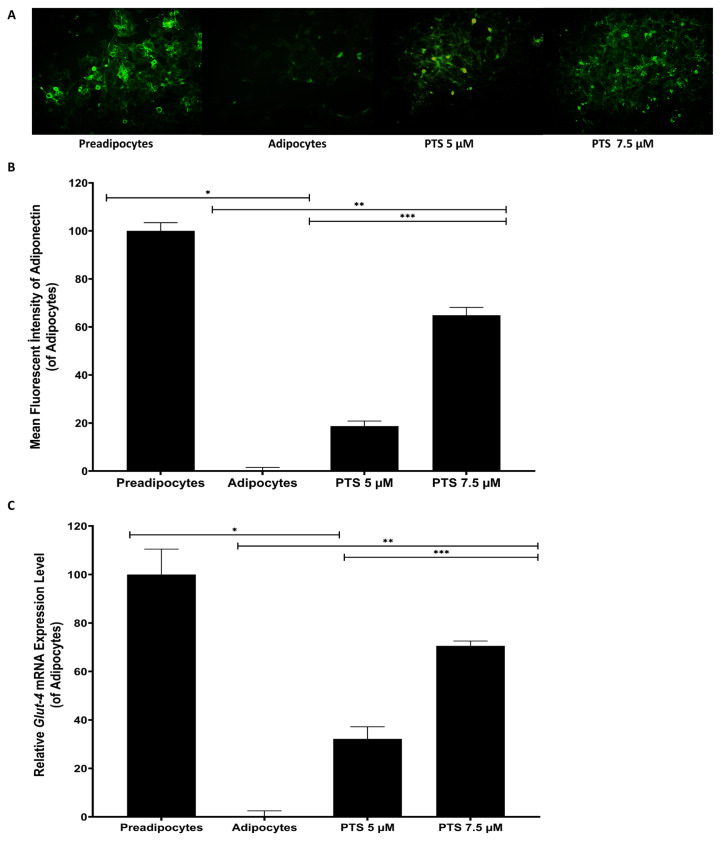
Immunofluorescence staining and RT-PCR data showing the effect of PTS on *Glut-4* expression. Mature adipocytes differentiated from preadipocytes for 14 days in the absence of PTS treatment or in the presence of different doses (5 −M or 7.5 −M) of PTS in adipocyte maintenance medium. Undifferentiated and untreated preadipocytes and differentiated and untreated adipocytes were used as controls. **A.** Representative phase-contrast microscopic images of cells stained with anti-Glut-4 antibody at 14th day at 10× magnification (scale bar: 200 −m). **B.** Quantitative data of relative intensity of anti-Glut-4 immunofluorescent staining. Coverslips from each cell group were evaluated. Six individual regions that randomly selected were imaged on each coverslip and the relative intensity of the immunofluorescent dye was measured using ImageJ software. Immunofluorescent staining analysis data were defined as relative variation of fluorescent signal intensities and mean ± SD (n = 3). **C.** Expression of the *Glut-4* gene at the mRNA level was determined by qRT-PCR, normalized with *Gapdh*. Data were analysed using the comparative Ct method from three independent experiments. Data with a relative change of Ct were presented as mean ± SD (n = 3) and expressed as fold change compared to adipocyte control. One-way ANOVA test was used for statistical analysis. *p < 0.05 and **p < 0.05 differ significantly from untreated 3T3-L1 adipocyte. ***p < 0.05 is significantly different from the 5 −M dose of PTS. PTS: pterostilbene; Glut-4: glucose transporter type 4; RT-PCR: quantitative reverse transcription PCR; Ct: cycle threshold, SD: standard deviation.

**Figure 9:**
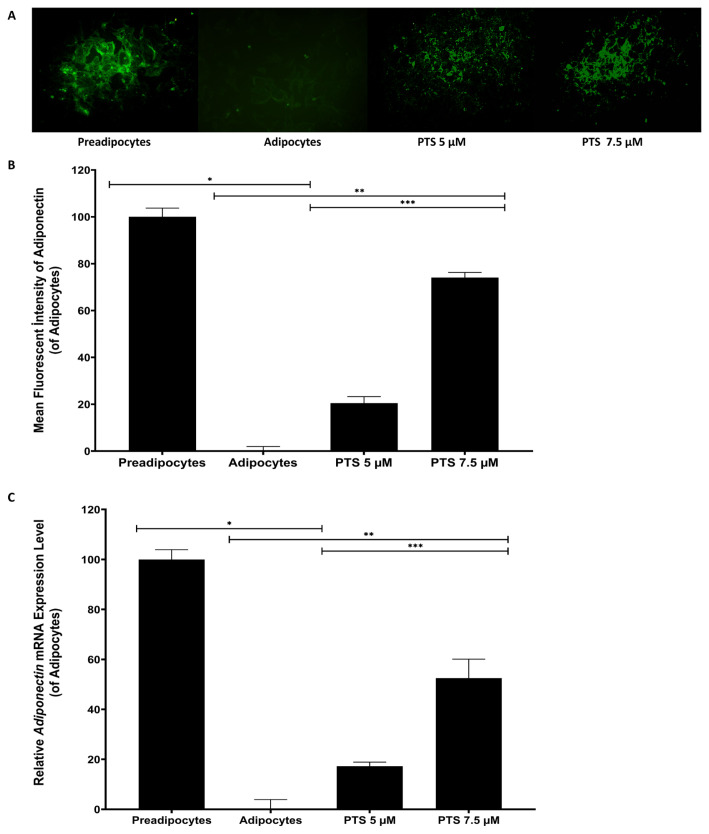
Immunofluorescence staining and RT-PCR data showing the effect of PTS on *Adiponectin* expression. Mature adipocytes differentiated from 3T3-L1 preadipocytes for 14 days in the absence of PTS treatment or in the presence of different doses (5 −M or 7.5 −M) of PTS in adipocyte maintenance medium. Undifferentiated and untreated preadipocytes and differentiated and untreated adipocytes were used as controls. The level of mRNA and intracellular expression of the *adiponectin* gene in cells was evaluated by immunofluorescent staining and qRT-PCR. **A.** Representative phase-contrast microscopic images of cells stained with antiadiponectin antibody at 14th days at 10× magnification (scale bar: 200 −m). **B.** Quantitative data of relative intensity of antiadiponectin immunofluorescent staining. Coverslips from each cell group were evaluated. Six individual regions that randomly chosen were imaged and the relative intensity of the immunofluorescent dye was measured using ImageJ software. Immunofluorescent staining analysis data are presented as the relative variation of fluorescent signal intensities and mean ± SD (n = 3). **C.** Expression of the *adiponectin* gene at the mRNA level was determined by qRT-PCR, normalized with *Gapdh*. Data were analysed using the comparative Ct method from three independent experiments. Data with a relative change of Ct were presented as mean ± SD (n = 3) and expressed as fold change compared to adipocyte control. One-way ANOVA test was used for statistical analysis. *p < 0.0001 and **p < 0.05 differ significantly from untreated 3T3-L1 adipocyte. ***p < 0.05 is significantly different from the 5 −M dose of PTS. PTS: pterostilbene; RT-PCR: quantitative reverse transcription PCR; Ct: cycle threshold, SD: standard deviation.

Preadipocytes were differentiated into mature adipocytes in an adipocyte maintenance medium in the presence or absence of PTS (5 −M and 7.5 −M) to investigate the effect of PTS on insulin resistance and adipogenesis, for 14 days. Glut-4 and adiponectin expression in cells at the 14th day of adipogenesis was evaluated by immunofluorescence staining and RT-PCR (Figures 8 and 9). In the study, undifferentiated and untreated preadipocytes and differentiated and untreated adipocytes were used as controls.

Glut-4 and adiponectin fluorescence was intensely observed in preadipocytes compared to adipocytes in immunofluorescent staining. However, the fluorescence intensity of both was dose-dependently increased in adipocytes treated with either 5 −M or 7.5 −M doses of PTS compared to untreated adipocytes. Moreover, the fluorescence intensity for Glut-4 and adiponectin was significantly increased in adipocytes treated with PTS 7.5 −M compared to the other treatment group (Figures 8A and 9A). Based on quantitative analysis of immunofluorescent staining, antibody fluorescence intensity for anti-Glut-4 and adiponectin increased 527.72% ± 16.44% and 537.21% ± 18.28% in preadipocytes compared to 3T3-L1 adipocytes, respectively (p < 0.0001 for all). The fluorescence intensity increased to 98.85% ± 10.03%, 342.41% ± 15.53% for Glut-4 and 109.94% ± 13.72%, 397.85% ± 10.80% for Adiponectin in adipocytes treated with 5 −M or 7.5 −M doses of PTS compared to untreated adipocytes. In addition, fluorescence intensity for Glut-4 and adiponectin increased by 122.48% and 137.14%, respectively (p < 0.0001 for all) in adipocytes treated with PTS 7.5 −M compared to the other treatment group (Figures 8B and 9B).

*Glut-4* mRNA expression was increased 299.00% ± 4.7% in untreated differentiated preadipocytes compared to adipocytes (p = 0.0002). *Glut-4* mRNA expression was increased 96.29% ± 22.00% and 210.9% ± 9% in adipocytes exposed with 5 −M or 7.5 −M doses of PTS compared to untreated adipocytes (p = 0.0005, p < 0.0001). On the other hand, *Glut-4* mRNA expression was increased by 58.43% in adipocytes treated with PTS 7.5 −M compared to the other treatment group (p = 0.0010) (Figure 8C). *Adiponectin* mRNA expression was increased 514.54% ± 10.03 in untreated differentiated preadipocytes compared to adipocytes (p < 0.0001).

*Adiponectin* mRNA expression was increased 88.91% ± 10.43% and 270.35% ± 19.99% in adipocytes treated with 5 −M or 7.5 −M doses of PTS, respectively, according to untreated adipocytes (p = 0.0046, p = 0.0017). However, *adiponectin* mRNA expression was increased by 96.04% in adipocytes treated with PTS 7.5 −M compared to the other treatment group (p = 0.0029) (Figure 9C).

## 4. Discussion

Obesity is one of the metabolic diseases and it has been characterized by excessive accumulation of fat in the body (Isganaitis and Lustig, 2005). Obesity is closely related to many diseases such as cancer, diabetes, cardiovascular, dyslipidemia, hypertension and sleep apnea. As a result, obesity has become a global public health problem. The high prevalence of obesity, together with its accompanying diseases, has led to increased interest in research on improving insulin sensitivity and inhibiting adipogenesis (Hurt et al., 2010; Raciti et al., 2018; Willis et al., 2021). 3T3-L1 preadipocytes are one of the cell models that allow the in vitro study of adipogenesis (Hsu et al., 2012; Raciti et al., 2018; Bedi et al., 2020; Derici et., 2021). In adipogenesis, increased insulin resistance, adipocyte hypertrophy, lipid accumulation and energy demand cause an increase in intracellular oxidative stress (Gregoire et al., 1998). Studies have reported that in the inhibition of preadipocyte differentiation, adipogenesis, lipid accumulation, as well as reduction of cell density, mitosis, and insulin resistance may be important mechanisms in inhibiting of adipocyte hypertrophy (Deepa et al., 2015; Balcazar et al., 2021). PTS is a natural phenolic compound found predominantly in blueberries (Aiyer et al., 2012). PTS has been reported as a natural, small molecular drug candidate for many chronic diseases with its pharmacokinetic properties, antioxidant properties, antiinflammatory activity, antidiabetic and anticancer capacity (Satheesh and Pari, 2006). Therefore, in our study, we focused on the antiobesogenic and antiadipogenic efficacy of PTS in 3T3-L1 preadipocytes by evaluating cell viability, cell proliferation, lipid accumulation, cell morphology, DNA condensation, *Glut-4* and *adiponectin* expression levels.

Our study showed that the addition of PTS to the medium decreased cell survival in 3T3-L1 preadipocytes in a time and dose-dependent manner, consistent with the literature (Hsu et al., 2012). In addition, treatment of 3T3-L1 preadipocytes with PTS appeared to reduce their viability in a time and dose-dependent manner, with IC_50_ values of 23.04 ± 1.89 −M at 24 h, 17.37 ± 1.22 −M at 48 h, and 14.60 ± 2.88 −M at 72 h. In contrast, at least approximately 90% of 3T3-L1 preadipocytes remained viable and had no cytotoxicity in treatment with 5 −M and 7.5 −M PTS. Therefore, these doses were applied to cells in further analyses to assess the effect of PTS on adipogenesis and differentiation. Moreover, live-dead staining data similarly showed that PTS treatment significantly reduced cell viability in 3T3-L1 adipocytes crosschecked to control, in a time and dose dependent manner. These results support that low doses of PTS may be an attractive cytotoxic agent. 3T3-L1 preadipocytes begin to differentiate by adipogenesis when they are exposed to adipogenic factors. Adipogenesis is a process characterized by cell growth arrest, mitotic clonal expansion, morphological changes and lipid accumulation (Guru et al., 2021). Studies have shown that many polyphenol compounds such as fucoxanthin, kaempferol, garcinol, resveratrol and PTS have inhibitory effectivenesses on lipid accumulation in 3T3- L1 adipocytes (Rayalam et al., 2008). In our study, the developed adipocytic phenotype was confirmed with staining the oil droplets. In addition, treatment of 3T3-L1 adipocytes to PTS at 5 −M and 7.5 −M concentrations decreased lipid accumulation by 30.64% ± 1.36% and 54.93% ± 3.14%, respectively, compared to the control. Therefore, PTS may be beneficial in preventing adipocyte hyperplasia by inhibiting adipogenesis via the stimulation of lipolysis in 3T3-L1 adipocytes.

Adipocyte culture differentiated from 3T3-L1 preadipocytes displays a monolayer of large, spherically shaped cells that partially overlap. Increased lipid synthesis and visible intracellular lipid vesicles in adipogenesis cause cells to become spheroid (Gregoire et al., 1998). In our study, the follow-up of Giemsa staining and F-actin staining using phase contrast microscopy, it was observed that PTS treatment caused a decrease in mitosis, cell density, integrity and fat droplets, an increase in cell size, a similar orientation of actin filaments in filamentous structure, and a fibroblastic shape with a long spindle-shaped structure in adipocytes compared to control, in a time and dose dependent manner. PTS may contribute to the loss of spherical morphology of cells and inhibit adipocyte hypertrophy by inducing lipolysis in adipocytes.

Apoptosis induction is a normal and healthy cellular process required to get rid of abnormal or expired cells without an inflammatory response. Nuclear chromatin condensation is the first morphological change observed during cell signalling apoptosis. In DAPI staining, it was observed that PTS induced nuclear chromatin condensation via apoptosis in 3T3-L1 adipocytes in a time and dose dependent manner, especially after 7 days of treatment. Thus, PTS exerts an antiadipogenic effect on 3T3-L1 adipocytes by inducing apoptosis and inhibiting differentiation. Preadipocytes differentiate into adipocytes upon growth arrest in adipogenesis, resulting in the formation of adipose tissue (Elmore, 2007). Therefore, the induction of apoptosis in differentiated adipocytes has been proposed as an important strategy for preventing and treating obesity (Rayalam et al., 2008). PTS treatment may prevent adipose tissue hyperplasia, as the induction of apoptosis may reduce the number of preadipocytes, causing them to differentiate into fewer adipocytes.

Adiponectin, an insulin-sensitizing adipocytokine, has been proven to decrease expression in 3T3-L1 adipocytes during adipogenesis (Fasshauer et al., 2002). Adiponectin plasma concentrations and fasting insulin concentrations have been shown to be adversely associated in in vivo studies (Yu et al., 2002). Therefore, induction of *adiponectin* expression is critical importance in the fight against obesity (Yamauchi et al., 2001). In a study, the protein and mRNA expression of the *adiponectin* gene decreased in 3T3-L1 adipocytes which differentiate in adipogenesis were observed (Hsu et al., 2012; Li et al., 2016). Consistent with the literature in this study, treatment of 3T3-L1 cells with PTS increased both mRNA and intracellular expression of *adiponectin* in a time and dose dependent manner. Our results support the suggestion that adiponectin induction via PTS increases Glut-4 production and translocation and shifts glucose metabolism (Ceddia et al., 2005).

Insulin stimulates glucose uptake in skeletal muscle and adipose tissue by mobilising Glut-4 to the cell membrane. Increased insulin resistance, weakened Glut-4 translocation, and decreased intracellular glucose uptake following insulin stimulation in obesity are a major defect (Szymczak-Pajor and Śliwińska, 2019). Therefore, some drugs used in the treatment of obesity target *Glut-4* with some genes and increase intracellular glucose uptake by promoting Glut-4 translocation in adipocytes with its upregulation (Kubota et al., 2006). A study also recently reported a remarkable decrease in Adiponectin production, *Glut-4* mRNA expression, translocation and glucose uptake in 3T3-L1 adipocytes in adipogenesis (Li et al., 2016).

In an in vivo study, it was reported that PTS treatment significantly increased Glut-4 protein expression in adipose tissue of diabetic rats (Sun et al., 2019). In our study, PTS treatment significantly up-regulated the mRNA and intracellular expression of the *Glut-4* gene during differentiation of 3T3-L1 adipocytes. Considering the roles of adiponectin and Glut-4 in adipogenesis and insulin resistance, as well as the antioxidant capacity of PTS, PTS may be an attractive phytochemical with the potential to regulate adipocyte differentiation in the fight against adipocytes hyperplasia according to these results.

## 5. Conclusion

Our data show that PTS inhibits cell proliferation and growth, reduces lipid accumulation, preserves fibroblastic morphology, and induces apoptosis in differentiated 3T3-L1 adipocytes. Also, according to our observations, PTS inhibited insulin resistance and the expression of adipogenic genes at mRNA and intracellular level in differentiated 3T3-L1 cells. These results show that PTS suppresses adipogenesis and reduces the differentiation of preadipocytes into mature adipocytes. Increased insulin resistance, increased preadipocyte proliferation and progressive adipogenesis are highly related to adipose tissue hyperplasia. Therefore, reducing adipocyte growth by suppressing the differentiation of preadipocytes and adipogenesis may be an alternative treatment strategy for obesity. All our results show that PTS can be considered to have a significant role in the treatment and prevention of obesity by reducing the differentiation of preadipocytes with its antiadipogenic and antiobesogenic effect.

## Figures and Tables

**Table:** The protocol real-time RT-PCR.

PCR step	Temperature (°C)	Time (min)	Cycle
Initial denaturation	94	3	1
Denaturation	94	0.5	35
Annealing	52–58	0.5	35
Extension	72	1	35
Final extension	72	5	1
